# Comparative Accuracy Analysis of the In Vitro Activity of Colistin Against Multidrug-Resistant (MDR) Non-Fermenters Using Broth Microdilution, Disk Elution, and Agar Dilution

**DOI:** 10.7759/cureus.85256

**Published:** 2025-06-02

**Authors:** Mitisha Soni, Deepti Chaurasia, Abhijit P Pakhare, Garima Kapoor

**Affiliations:** 1 Microbiology, Mahaveer Institute of Medical Science, Bhopal, IND; 2 Microbiology, Gandhi Medical College, Bhopal, IND; 3 Community and Family Medicine, All India Institute of Medical Sciences, Bhopal, IND

**Keywords:** agar dilution test, antimicrobial susceptibility testing, broth microdilution, colistin broth disc elution, gold standard, multidrug-resistant, non-fermenters

## Abstract

Objectives: Colistin is a crucial treatment approach for multidrug-resistant (MDR) gram-negative bacterial infections but its susceptibility testing is challenging for clinical laboratories. Therefore, the study was planned to evaluate the diagnostic accuracy of the colistin broth disk elution (CBDE) method and the colistin agar test (CAT) in determining colistin minimum inhibitory concentrations (MICs) among MDR non-fermenting gram-negative bacilli (NF-GNB). The performance of these methods is compared to the gold standard broth microdilution (BMD) technique, with the goal of identifying reliable, resource-feasible alternatives for routine clinical laboratory use.

Material and methods: A total of 74 MDR non-fermenter isolates (37 *Pseudomonas aeruginosa*, 24 *Acinetobacter baumannii* complex, eight *Stenotrophomonas maltophilia*, three *Elizabethkingia meningoseptica*, and two *Chryseobacterium indologenes*) were tested by BMD in polystyrene microtiter plates. CBDE and CAT were performed and deciphered as per Clinical and Laboratory Standards Institute (CLSI) interpretive criteria (M100; 32nd Edition).

Statistical analysis: The diagnostic performance characteristics of CBDE and CAT were assessed by comparing them to the gold standard BMD. Agreement statistics, including categorical agreement (CA), sensitivity, specificity, positive predictive value (PPV), and negative predictive value (NPV), were calculated. Kappa statistics with 95% CI were used to determine the level of agreement.

Results: CBDE and CAT showed CAs of 90.5% and 85.1%, respectively, with respect to BMD. The overall sensitivity, specificity, PPV, and NPV for CBDE, with reference to BMD, were 88.8%, 91.4%, 85.7%, and 93.4%, respectively, while for CAT, these were 88.8%, 82.9%, 75%, and 92.8%, respectively. Agreement statistics (kappa) for CBDE was 0.79 (95% CI: 0.56 to 1), and for CAT, it was 0.69 (95% CI: 0.46 to 0.97), both indicating substantial agreement. Species-wise stratified analyses for *Pseudomonas* and *Acinetobacter* showed that the diagnostic properties and kappa values were better for *Pseudomonas* species with both CBDE and CAT.

Conclusion: Various susceptibility testing methods for colistin give diverse results. CBDE can be used as an alternative to BMD for *Pseudomonas aeruginosa* in a constrained resource setting.

## Introduction

Polymyxins are a re-emerging class of antimicrobials that act as surface active agents against most gram-negative bacteria (GNB), including multidrug-resistant (MDR) ones [1]. Polymyxin B and polymyxin E (colistin) are the main polymyxin agents being arrayed in medical practice. Colistin is required to manage gram-negative infections that are resistant to anti-*Pseudomonal* penicillins, aminoglycosides, quinolones, cephalosporins, monobactams, and carbapenems. Thus, it is being employed as the ultimate refuge drug for managing life-endangering infections [2]. Progressively, strains of *Acinetobacter baumannii *and *Pseudomonas aeruginosa* that exhibit resistance to most available antibiotics, except polymyxins, have become the primary etiology of healthcare-associated infections (HAIs) in critically ill patients [3].

In the current era of antibiotic resistance, physicians have resumed using colistin despite its toxicity [4]. Although sufficient data are now available that make the clinical and pharmacokinetic/pharmacodynamic (PK/PD) efficacy of this agent debatable [5,6], its rampant use continues, either due to a dearth of alternative active antimicrobials (e.g., metallo-β-lactamase producers) or the inaccessibility and high cost of effective agents against MDR infections [7]. As a consequence, the emergence of colistin-resistant bacteria, although reported sporadically to date, is becoming a clinical concern [4].

Disk diffusion assays have questionable reliability for polymyxins because these antibiotics diffuse poorly into agar. Therefore, no definitive correlation can be drawn between zone diameters and minimum inhibitory concentrations (MICs). The Clinical and Laboratory Standards Institute (CLSI) has issued polymyxin susceptibility testing guidelines using dilution methods against *Pseudomonas aeruginosa*, *Acinetobacter* spp., and several other non-fermenting gram-negative bacilli (NF-GNB) [8]. There is a lack of sufficient true resistance data for this group of antibiotics among non-fermenters (NFs), as relatively few surveys have been conducted. The recent emergence of colistin resistance due to the mcr-1 plasmid has further complicated susceptibility testing for this drug [9].

Due to the absence of clinical and PK/PD evidence supporting any specific colistin MIC value as “susceptible” for P*seudomonas aeruginosa* and *Acinetobacter* spp., the CLSI has, since 2019, provided only “intermediate” and “resistant” interpretive categories, with no “susceptible” classification for colistin [7].

Considering the increasing use of colistin and the relative lack of awareness regarding resistance mechanisms, this study was planned to systematically evaluate two MIC-based methods for colistin susceptibility testing: the colistin broth disk elution (CBDE) method and the colistin agar test (CAT), in accordance with CLSI guidelines (M100; 32nd Edition) [8]. The purpose of evaluating these tests was not to develop a more accurate method per se but rather to develop a method as accurate as broth microdilution (BMD) that is more suitable for routine clinical laboratory use, i.e., one that utilizes readily available materials and is of low complexity to execute [7].

We expect that the findings of this research will provide guidance to diagnostic laboratories in comparing simplified and CLSI-recommended testing methods against gold-standard techniques for antibiotics with complex phenotypic testing profiles.

## Materials and methods

Study design

This concurrent cohort study was conducted over 18 months (January 2021 to July 2022) in the Department of Microbiology at a tertiary care teaching hospital in central India. A waiver of informed consent was granted by the institutional ethics committee, as de-identified samples were used, posing minimal risk to patient privacy (27088/MC/IEC/2021). Various clinical samples were processed to isolate and identify MDR NF-GNB using standard microbiological techniques. Susceptibility testing, including colistin susceptibility via BMD (gold standard), CBDE, and CAT, was performed in accordance with CLSI guidelines (M100; 32nd Edition) [8,10]. All discordant results (major errors (MEs) and very major errors (VMEs)) were retested in parallel with BMD to confirm accuracy. For BMD, an in-house* Pseudomonas aeruginosa* (MIC >16 µg/mL) served as the positive control, while *Pseudomonas aeruginosa* ATCC 27853 (MIC = 0.5-4 µg/mL) served as the negative control. MDR isolates were defined as those resistant to three or more antibiotic classes [11]. Colistin-resistant strains identified by BMD were further screened for plasmid-mediated resistance genes (mcr-1, mcr-2, mcr-3) via polymerase chain reaction (PCR) as described by Al-Kadmy et al. [12].

Colistin broth microdilution

**Figure 1:**
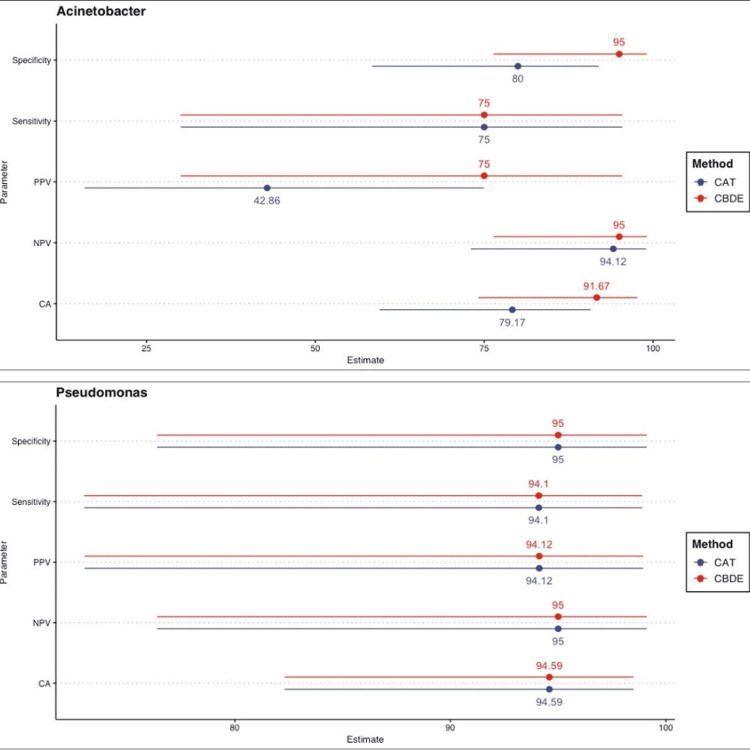
Genus-wise stratified analysis of diagnostic properties for Pseudomonas spp. and Acinetobacter spp. CBDE, colistin broth disk elution; CAT, colistin agar test

Initially, 50 μL of cation-adjusted Mueller-Hinton broth (CAMHB, pH 7.2-7.4) was dispensed into sterile, round-bottom 96-well microplates. The potency of colistin sulfate powder (30 UN/µg) was used to prepare a primary stock solution with a final concentration of 1 mg/mL active colistin sulfate [10]. A working stock was prepared from the primary solution. The working stock was mixed with a standardized bacterial inoculum (0.5 McFarland) to generate two-fold serial dilutions of colistin ranging from 0.03 µg/mL to 16 µg/mL. Each plate contained seven isolates, including the quality control strain *Pseudomonas aeruginosa* ATCC 27853, while the 8th row served as the growth control (Figure 1).

These microtiter plates were incubated at 35 ± 2°C for 16 to 20 h in an ambient air incubator within 15 minutes of adding the inoculum. Finally, MIC was read as the lowest concentration of colistin that completely inhibited the growth of the organism in the microdilution wells, as detected by the unaided eye.

Colistin broth disk elution

This is a tube dilution method using 10 μg colistin sulfate disks in CAMHB (10 mL tubes) to attain end levels: 0 μg/mL (growth control), 1 μg/mL, 2 μg/mL, and 4 μg/mL colistin.

For each isolate to be tested, four tubes of CAMHB (10 mL) were labeled as 1 μg/mL, 2 μg/mL, 4 μg/mL, and control. One, two, and four colistin discs were added aseptically to the tubes marked 1 μg/mL, 2 μg/mL, and 4 μg/mL, respectively. Then the tubes were vortexed and rested for 30 minutes at room temperature to allow colistin to elute from the disks.

Then, 50 μL bacterial inoculum (0.5 McFarland) was added to the control and 1 μg/mL, 2 μg/mL, and 4 μg/mL tubes. Purity subculture was done from the original inoculum tube to a blood agar plate. All the inoculated tubes were vortexed after capping. The tubes and purity plate were incubated at 35°C in ambient air for 16-20 hours. The caps were loosened slightly before incubation.

The growth control tube and the purity plate were examined to ensure the purity of the inoculum. The MIC was marked as the lowest concentration that completely inhibited the isolate under test. Although, as per CLSI, the only approved method for colistin MIC determination in *Acinetobacter* spp. is BMD, we used the following criteria for *Pseudomonas aeruginosa* and all other NFs: ≤2 μg/mL = intermediate and ≥4 μg/mL = resistant.

Colistin agar dilution test

This is an agar dilution technique using Mueller Hinton Agar (MHA) with final concentrations of colistin sulfate as: 0 μg/mL (growth control), 1 μg/mL, 2 μg/mL, and 4 μg/mL colistin.

Each colistin agar plate with increasingly doubled dilutions of colistin was divided to test up to 10 isolates per plate. Using a pipette, 10 μL of the 1:10 dilution of 0.5 McFarland bacterial inoculum was taken and streaked onto the appropriate part of each colistin agar plate.

The colistin agar plates and blood agar purity plate were incubated in ambient air overnight at 35°C. The purity plate was examined to ensure the inoculum was pure, and the growth control plate was also examined.

The MIC was determined using the colistin plates under transmitted light as the lowest concentration of colistin that completely inhibited the growth of the test strain. Although, as per CLSI, the only approved method for colistin MIC determination in *Acinetobacter* spp. is BMD, we used the following criteria for *Pseudomonas aeruginosa* and all other NFs: ≤ 2 μg/mL = intermediate and ≥ 4 μg/mL = resistant.

Statistical analysis

Statistical analyses were performed using R (version 4.2.2) and OpenEpi (web-based epidemiological calculator). The diagnostic performance characteristics of CBDE and CAT were assessed by comparing them to the gold standard BMD. Agreement statistics, including CA, sensitivity, and specificity, were calculated. Kappa statistics with 95% confidence intervals were used to determine the level of agreement. Also, genus-specific analyses were conducted for Pseudomonas and Acinetobacter to evaluate diagnostic properties and agreement statistics.

We did not calculate diagnostic test properties for *Stenotrophomonas maltophilia* (n = 8), *Elizabethkingia meningoseptica* (n = 3), and *Chryseobacterium indologenes* (n = 2) because of the very small sample size. Visualization summarizing diagnostic properties of CBDE and CAT was created using point-range diagrams, where the point indicates an estimate while the range indicates 95%CI.

The breakpoint results derived from the aforementioned methods were interpreted in line with the CLSI guidelines [8] and then collated with those retrieved using the BMD reference method. An intermediate result by rBMD but resistant by CBDE or CAT was termed an ME, and it was classified as a VME when the result was resistant by rBMD but intermediate by CBDE or CAT, as per Humphries et al. [7]. The percentage of identical results in reference and test methods gives the CA. Sensitivity identified true positives, and specificity measured true negatives [13]. To check test reliability, we measured inter-rater agreement for categorical items using Cohen’s Kappa (CK) statistics; where a Kappa value of 0-0.20 indicates no agreement, 0.21-0.39 minimal agreement, 0.40-0.59 weak agreement, 0.60-0.79 moderate agreement, 0.80-0.90 strong agreement, and >0.90 denotes almost perfect agreement [14].

We also performed genus-wise stratified analyses for *Pseudomonas* and *Acinetobacter *spp.

## Results

The quality control of colistin MIC for P. aeruginosa ATCC 27853 was within the assigned range (0.5 to 4 µg/mL for colistin) across all three methods. Among 74 NF isolates tested for colistin MIC using BMD, 47 (63.5%) were colistin intermediate-sensitive, and 27 (36.5%) were colistin-resistant (Table 1).

**Table 1:** Colistin MIC results for NFs using three CLSI-recommended methods (M100; 32nd edition). I, intermediate; R, resistant; NA, not available; P, pus isolate; U, urine; B, blood; F, fluid; BMD, broth microdilution; CBDE, colistin broth disc elution; CAT, colistin agar dilution test; NFs, non-fermenters

Species (number of isolates)	Genotype	Reference BMD (µg /mL)	CBDE (µg /mL)	CAT (µg /mL)
Isolates with acquired colistin resistance				
*Pseudomonas aeruginosa*, n = 17				
U-1827	mcr-1,2,3 negative	8 (R)	>4 (R)	>4 (R)
B-1740		16 (R)	>4 (R)	>4 (R)
B-2053		8 (R)	>4 (R)	>4 (R)
F-659		4 (R)	4 (R)	4 (R)
U-1847		16 (R)	>4 (R)	>4 (R)
U-2409		8 (R)	>4 (R)	>4 (R)
P-2546		4 (R)	4 (R)	4 (R)
P-2738		8 (R)	>4 (R)	>4 (R)
B-2807		16 (R)	>4 (R)	>4 (R)
B-3911		16 (R)	>4 (R)	>4 (R)
U-285		64 (R)	>4 (R)	>4 (R)
F-94		4 (R)	4 (R)	4 (R)
U-544		8 (R)	2 (I)	4 (R)
P-628		16 (R)	>4 (R)	2 (I)
P-2440		64 (R)	>4 (R)	>4 (R)
B-2448		8 (R)	>4 (R)	>4 (R)
B-995		8 (R)	>4 (R)	>4 (R)
*Acinetobacter baumanii,* n = 4				
U-4443	mcr-1,2,3 negative	16 (R)	4 (R)	2 (I)
P-471		8 (R)	>4 (R)	>4 (R)
B-594		4 (R)	4 (R)	4 (R)
B-3780		4 (R)	2 (I)	4 (R)
*Stenotrophomonas maltophilia,* n = 2				
B-422	mcr-1,2,3 negative	16 (R)	4 (R)	4 (R)
P-903		64 (R)	4 (R)	4 (R)
*Elizabethkingia meningoseptica,* n = 2				
B-667	mcr-1,2,3 negative	16 (R)	4 (R)	4 (R)
B-1030		64 (R)	2 (I)	4 (R)
*Chryseobacterium indologenes, *n = 2				
B-1100	mcr-1,2,3 negative	64 (R)	4 (R)	4 (R)
B-1300		16 (R)	4 (R)	2 (I)
Colistin intermediate susceptible “I” isolates				
*Pseudomonas aeruginosa,* n = 20				
U-1198	NA	2 (I)	2 (I)	2 (I)
P-1574		0.5 (I)	1 (I)	1 (I)
U-2053		0.125 (I)	1 (I)	1 (I)
P-1436		0.25 (I)	1 (I)	1 (I)
P-1609		2 (I)	2 (I)	2 (I)
P-1805		0.06 (I)	1 (I)	1 (I)
P-1740		0.25 (I)	1 (I)	1 (I)
P-2684		0.5 (I)	1 (I)	1 (I)
B-2814		0.125 (I)	1 (I)	1 (I)
B-2261		2 (I)	2 (I)	4 (R)
P-267		2 (I)	4 (R)	1 (I)
P-282		0.125 (I)	1 (I)	1 (I)
B-5156		0.06 (I)	1 (I)	1 (I)
B-561		0.5 (I)	1 (I)	1 (I)
P-594		0.25 (I)	1 (I)	1 (I)
U-887		2 (I)	2 (I)	2 (I)
P-712		2 (I)	2 (I)	2 (I)
B-2473		1 (I)	1 (I)	1 (I)
B-3318		2 (I)	2 (I)	2 (I)
U-2479		1 (I)	1 (I)	1 (I)
*Acinetobacter baumannii,* n = 20				
P-1867	NA	0.5 (I)	2 (I)	4 (R)
P-1650		0.125 (I)	1 (I)	1 (I)
B-1248		2 (I)	2 (I)	4 (R)
B-2638		1 (I)	1 (I)	1 (I)
B-1967		0.06 (I)	1 (I)	1 (I)
F-643		0.5 (I)	1 (I)	1 (I)
U-1876		2 (I)	2 (I)	4 (R)
U-3612		2 (I)	4 (R)	2 (I)
P-4097		0.125 (I)	1 (I)	1 (I)
B-3733		0.5 (I)	1 (I)	1 (I)
P-429		2 (I)	2 (I)	4 (R)
B-2639		1 (I)	1 (I)	1 (I)
B-1968		0.06 (I)	1 (I)	1 (I)
F-641		0.5 (I)	1 (I)	1 (I)
P-2097		0.125 (I)	1 (I)	1 (I)
B-1733		0.5 (I)	1 (I)	1 (I)
P-4096		0.125 (I)	2 (I)	1 (I)
B-3731		0.5 (I)	1 (I)	1 (I)
P-3097		0.125 (I)	1 (I)	2 (I)
B-2733		0.5 (I)	1 (I)	1 (I)
*Stenotrophomonas maltophilia,* n = 6				
B-479	NA	0.5 (I)	1 (I)	2 (I)
P-539		0.25 (I)	1 (I)	2 (I)
F-653		2 (I)	4 (R)	4 (R)
B-1937		0.125 (I)	2 (I)	2 (I)
P-436		0.5 (I)	4 (R)	4 (R)
F-535		2 (I)	2 (I)	2 (I)
*Elizabethkingia meningoseptica,* n = 1				
F-363	NA	0.5 (I)	2 (I)	4 (R)

Among *Pseudomonas aeruginosa-*resistant isolates (by the reference BMD method), one isolate was classified as intermediate susceptible by CBDE (VME = 1/17), and another isolate was intermediate susceptible by CAT (VME = 1/17). Among the colistin intermediate susceptible isolates, one isolate each showed a resistant MIC by CBDE and CAT (ME = 1/20). For colistin-resistant *Acinetobacter baumannii *complex, one isolate each was intermediate susceptible by CBDE and CAT (VME = 1/4). Among colistin intermediate susceptible isolates, one isolate showed a resistant result by CBDE (ME = 1/20), and four isolates showed resistant results by CAT (ME = 4/20) (Table 2).

**Table 2:** Summary of test performance of CBDE and CAT methods compared to the reference BMD. I, intermediate; R, resistant; BMD, broth microdilution; CBDE, colistin broth disc elution; CAT, colistin agar dilution test; VME, very major error; ME, major error

Test results	CBDE	CAT
*Pseudomonas aeruginosa*, n = 37 tests (I = 20, R = 17)		
No. of VME	1/17 (5.8%)	1/17 (5.8%)
No. of ME	1/20 (5%)	1/20 (5%)
*Acinetobacter baumannii*, n = 24 tests (I = 20, R = 4)		
No. of VME	1/4 (25%)	1/4 (25%)
No. of ME	1/20 (5%)	4/20 (20%)
*Stenotrophomonas maltophilia*, n = 8 tests (I = 6, R = 2)		
No. of VME	0/2 (0%)	0/2 (0%)
No. of ME	2/6 (33.3%)	2/6 (33.3%)
*Elizabethkingia meningoseptica*, n = 3 tests (I = 1, R = 2)		
No. of VME	1/2 (50%)	0/2 (0%)
No. of ME	0/1 (0%)	1/1 (100%)
*Chryseobacterium indologenes*, n = 2 tests (I = 0, R = 2)		
No. of VME	0/2 (0%)	1/2 (50%)
No. of ME	0/0 (0%)	0/0 (0%)

**Figure 2:**
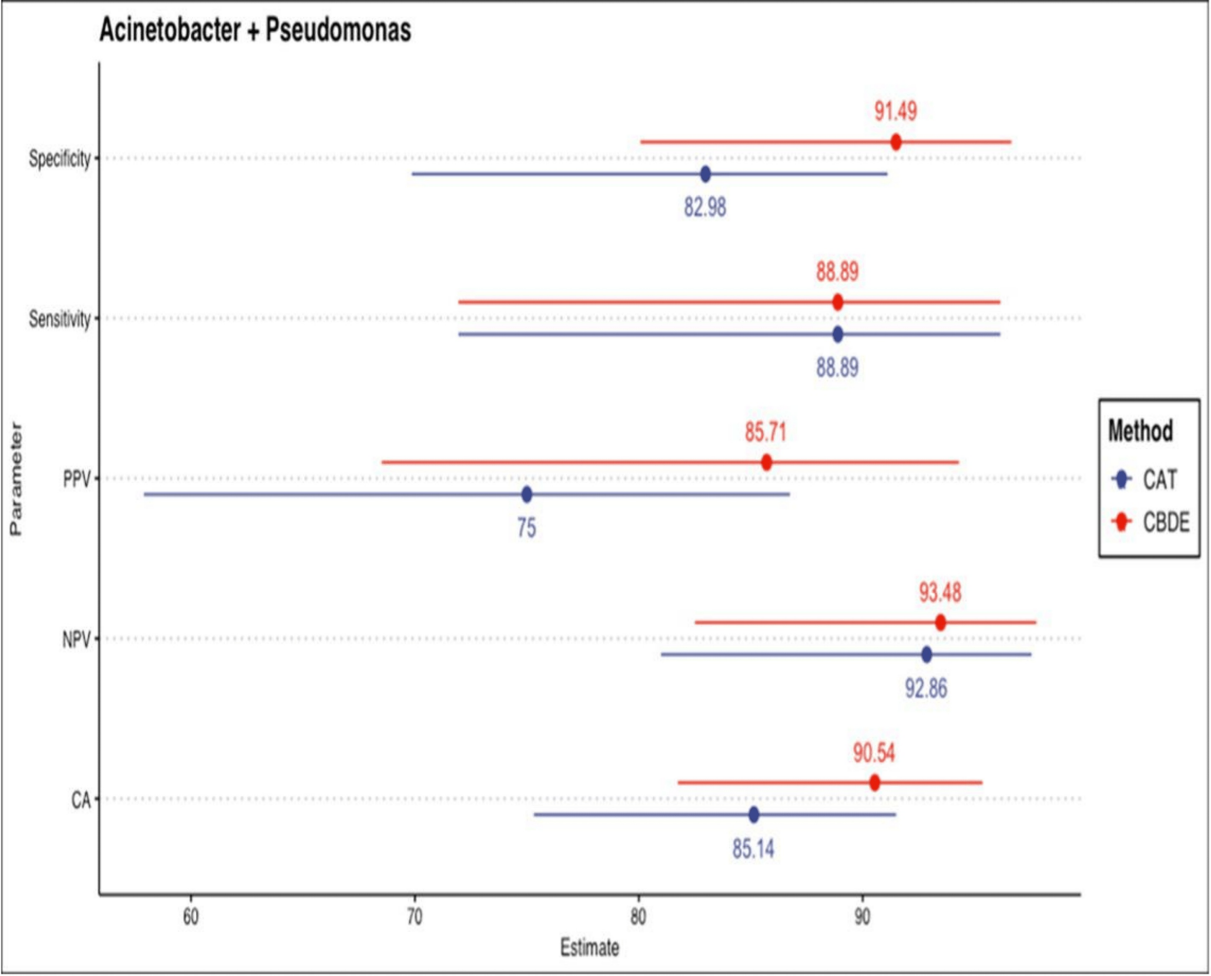
Point-range diagram summarizing the diagnostic properties of CBDE and CAT. Points represent estimates, and lines indicate 95% CI. CBDE, colistin broth disk elution; CAT, colistin agar test

CBDE and agar dilution methods showed CA of 90.5% and 85.1%, respectively, compared to BMD for 61 *Acinetobacter* and *Pseudomonas* strains. Overall sensitivity, specificity, PPV, and NPV for CBDE relative to BMD were 88.8%, 91.4%, 85.7%, and 93.4%, respectively. For CAT, these values were 88.8%, 82.9%, 75%, and 92.8%, respectively. Agreement statistics (kappa) were 0.79 (95% CI: 0.56 to 1) for CBDE and 0.69 (95% CI: 0.46 to 0.97) for CAT, both indicating substantial agreement (Figure 1). Genus-wise stratified analyses for *Pseudomonas* spp. and *Acinetobacter* spp. showed that diagnostic performance and agreement were better for Pseudomonas spp. by both CBDE (k = 0.89) and CAT (k = 0.89), whereas agar dilution showed low agreement (k = 0.42) (Figure 2).

## Discussion

In current medical practice, NF-GNB accounts for 12-15% of all bacterial strains [15]. The prevalence of carbapenem and colistin resistance in NFs has been reported to be as high as 70% and 6.5%, respectively, among HAIs in Indian ICUs [16].

Polymyxins are the last-resort antimicrobial agents for MDR infections. However, their PK/PD and clinical data indicate limited effectiveness, and alternative agents are strongly recommended [17]. Considering the widespread use of colistin throughout the country for treating HAIs, and the ongoing unregulated use of colistin in livestock for treatment, prevention, and growth promotion [18], colistin MIC testing cannot be deferred. Otherwise, irrational empirical use of colistin will significantly contribute to drug resistance in low-resource settings.

Different antimicrobial susceptibility testing methods for colistin vary greatly in their results [19]. Disk diffusion and gradient diffusion methods are not recommended due to high error rates [20], yet some laboratories continue using them because of budget constraints and the easy availability of required supplies. As an alternative, laboratories may outsource colistin MIC testing, but extended turnaround times can cause significant delays in initiating effective treatment for critically ill patients. The acceptable methods for colistin MIC testing are BMD, CBDE, and agar dilution methods. CBDE serves as a practical, accurate, and reproducible choice for colistin testing in laboratories of various sizes, using readily available and affordable supplies [20]. CAT requires substantial technologist time for setup (special media - colistin agar plate) but can test up to 10 isolates at once [8,20]. The robustness of BMD is limited by the need for dedicated staff with good pipetting skills and precise digital weighing equipment. Hence, this gold standard for NFs is plagued by complexity. Furthermore, other methods have yielded inaccurate results for *Acinetobacter* spp. and none for other NFs when evaluated by CLSI [8]. Therefore, we evaluated two MIC-based, CLSI-recommended alternative methods for colistin MIC testing: CBDE and CAT. The rationale for this evaluation was to identify a method as accurate as BMD, but more suitable for routine clinical laboratory use, i.e., one that uses accessible materials and is less complex to perform.

There are very few studies on susceptibility testing for NFs, despite widespread concern [21]. One study revealed high error rates for NFs (ME: 33.33%; VME: 12.5%), though it included only nine *Acinetobacter baumannii* isolates [22]. Another study on 106 *Acinetobacter baumannii* isolates showed a ME rate of 3.3% and a VME rate of 5.6% [7]. In contrast, research from two U.S.-based microbiology laboratories found 100% essential agreement (EA) and categorical agreement (CA) for both *Pseudomonas aeruginosa* and *Acinetobacter baumannii* [20]. An Indian study (including 100 susceptible and 25 resistant *Acinetobacter baumannii* isolates) evaluated the CBDE method against the gold standard BMD and found CA, EA, sensitivity, and specificity of 98.4% (n = 123), 97.6% (n = 122), 100%, and 98.4%, respectively. The ME rate was 1.6% (n = 2), with no VMEs [21]. In our study, among 13 isolates with MIC 2 µg/mL by the gold standard, three were falsely detected as resistant to CBDE. Simner et al. [20] used mcr-1-producing strains with MICs of 2 µg/mL and 4 µg/mL and observed VMEs using the CBDE method. The discrepancy may be due to disks from different manufacturers and the unverified concentration of colistin eluted in CA-MHB. All these studies discuss plasmid-mediated colistin resistance through mcr genes and susceptibility testing using CBDE, though CBDE is not approved by CLSI for *Acinetobacter baumannii*. In this context, we suggest that for *Acinetobacter baumannii*, where mutations are the major resistance mechanism [23], CBDE may be useful, showing substantial agreement with BMD (kappa = 0.79, 95% CI: 0.56-1), especially in resource-constrained settings, compared to not performing susceptibility testing for colistin at all.

Recently, CLSI [8] also recommended CAT, an adaptation of agar dilution, for detecting colistin MIC in *Pseudomonas aeruginosa* and Enterobacterales. A study by Singhal et al. [19] showed moderate overall agreement of CAD with BMD (CA < 90%). A study with 11 *Acinetobacter baumannii* isolates showed 80% agreement between CAD and BMD using polysorbate 80 plates [24], while another reported 55% EA using tissue culture-coated plates and 20 *Acinetobacter baumannii* isolates [25]. A separate study with only seven *Acinetobacter baumannii* isolates reported high agreement between BMD and AD for both colistin and polymyxin B [26], but generalizing such limited findings is not appropriate. Though the MICs obtained with CAD in the present study were consistent with BMD, a previous study reported higher MICs using AD [27]. A study by Shams N et al. [28] from Qatar demonstrated 90.48% CA between CAD and BMD, with a kappa value of 0.79. In our study, the agar dilution method showed 85.1% CA with BMD. Agreement statistics (kappa) for CAT was 0.69 (95% CI: 0.46-0.97), indicating substantial agreement. This might be due to the limited number of NFs included in previous studies (e.g., only two *Pseudomonas aeruginosa* in Shams N et al. [28]). Genus-wise stratified analysis showed better diagnostic performance and agreement for *Pseudomonas* spp. using both CBDE (CK = 0.89) and CAT (k = 0.89), while agar dilution showed low agreement for *Acinetobacter* spp. (k = 0.42). Escalante et al. [29] suggested adding an ethylenediaminetetraacetic acid (EDTA)-supplemented colistin plate to CAT (as EDTA is a known mcr-1 inhibitor) for accurate detection of mcr-1 producers.

High VME rates in *Acinetobacter* spp. may be due to the low number of colistin-resistant isolates (n = 4, 25%), which can exaggerate VME rates even with a few false-susceptible results [30]. Overall, CBDE showed better sensitivity, specificity, PPV, and NPV than CAT when compared with BMD.

So far, 10 mobile mcr genes have been described [6,11,12]. In our study, all colistin-resistant strains (as determined by BMD) were subsequently tested using PCR for plasmid-mediated colistin-resistance genes (mcr-1, mcr-2, mcr-3). However, negative molecular test results for these genes do not exclude the possibility of novel mcr genes or chromosomal resistance mechanisms [28].

Moreover, to avoid bias, the inclusion of both susceptible and resistant strains is essential [28]. Therefore, the bacterial strain panel selected for this study included both types. Although the culture isolates were prospectively collected during the study period, they were screened for colistin resistance using BMD and then stored before inclusion in the study. This approach helped increase the number of colistin-resistant isolates.

Limitations

All testing procedures used antibiotic discs and culture media from a single manufacturer. Also, subculturing and frozen storage may alter colistin MICs, as reported in various studies. We excluded isolates showing skipped wells from the analysis. Laboratories should carefully repeat tests for strains showing skipped dilutions by both CBDE and CAD methods.

Finally, we did not perform a complete genotypic analysis of colistin resistance mechanisms.

## Conclusions

The need for a refined strategy for colistin MIC determination is urgent, as we observe a rising prevalence of MDR gram-negative bacterial infections with limited therapeutic options. A larger multicenter study using the same isolates and a greater number of resistant non-fermenters tested across multiple sites is required to validate the accuracy and reproducibility of different colistin MIC testing methods. Further evaluation of diverse non-fermenter isolates with varied MICs and different resistance mechanisms would better address this concern.

While CLSI has progressively improved various techniques to facilitate colistin testing, including breakpoints and test methods, the limitations of this drug should be carefully considered prior to its use, especially when other antimicrobial agents with well-documented efficacy are available.
